# Numerical Simulation on Open Wellbore Shrinkage and Casing Equivalent Stress in Bedded Salt Rock Stratum

**DOI:** 10.1155/2013/718196

**Published:** 2013-10-01

**Authors:** Jianjun Liu, Linzhi Zhang, Jinzhou Zhao

**Affiliations:** ^1^State Key Laboratory of Oil and Gas Reservoir Geology and Exploitation, Southwest Petroleum University, Chengdu, China; ^2^School of Civil Engineering and Architecture, Southwest Petroleum University, Chengdu, China

## Abstract

Most salt rock has interbed of mudstone in China. Owing to the enormous difference of mechanical properties between the mudstone interbed and salt rock, the stress-strain and creep behaviors of salt rock are significantly influenced by neighboring mudstone interbed. In order to identify the rules of wellbore shrinkage and casings equivalent stress in bedded salt rock stratum, three-dimensional finite difference models were established. The effects of thickness and elasticity modulus of mudstone interbed on the open wellbore shrinkage and equivalent stress of casing after cementing operation were studied, respectively. The results indicate that the shrinkage of open wellbore and equivalent stress of casings decreases with the increase of mudstone interbed thickness. The increasing of elasticity modulus will reduce the shrinkage of open wellbore and casing equivalent stress. Research results can provide the scientific basis for the design of mud density and casing strength.

## 1. Introduction

The salt rock has obvious creep behavior under high-temperature and high-pressure situation in deep stratum. Down-hole accidents such as open wellbore shrinkage, drill pipe sticking, and casing collapse happened in Tarim oilfield, Jianghan oilfield and Shengli oilfield before, which caused huge economic loss [1–3]. Scholars have carried out considerable research on wellbore shrinkage and casing collapse in salt rock stratum. Yang et al. and Zhao studied the wellbore shrinkage in salt rock and proposed the nonlinear creep model [4, 5]. Besides, Willson et al. studied the casing extrusion force in creep stratum [6–14]. Most salt rock is bedded salt rock in China, and the mudstone interbed has the features of large quantity and small single layer thickness. There is a huge difference of mechanical properties between the mudstone interbed and salt rock. The stress-strain distribution and creep behavior of salt rock are significantly influenced by neighboring mudstone interbed. Wang et al. studied the mechanical properties of bedded salt rock [15–17]. 

The studies on wellbore shrinkage and equivalent stress on casing in bedded salt rock stratum ignored the influence of mudstone interbed, which has great deviations with actual situation of bedded salt rock stratum. It is necessary to study the wellbore shrinkage and equivalent stress on casings in salt rock stratum considering the influence of mudstone interbed within salt rock. Three-dimensional finite difference models were established to study the influence brought by mudstone interbed. The effects of thickness and elasticity modulus of mudstone interbed were discussed, respectively.

## 2. The Mathematical Model of Wellbore Shrinkage and Equivalent Stress on Casing in Bedded Salt Rock Stratum

According to the stress state and temperature situation, the salt rock takes different creep mechanisms. The salt rock in the research is located in 2000 m underground. The salt rock has high stress and relatively low temperature (lesser than 250°C), and the creep deformation mainly comes from dislocation slip. At this point, the creep behavior follows the creep rule of Norton Power:

(1) $$\begin{array}{c} {\dot{\varepsilon }}_{\text{cr}}=Aq^{n},\\ q=\sqrt{3J_{2}},\\ J_{2}=\frac{1}{2}S_{ij}S_{ij}, \end{array}$$

where ${\dot{\varepsilon }}_{\text{cr}}$ is steady creep ratio, *A*, *n* are material parameters, *S*_*ij*_ is the stress deviator.

Referring to other scholar's test, the material creep parameters of salt rock and mudstone interbed are shown in Table 1 [18].

Suppose the deformation of cement ring and casing follows the elastic constitutive model. According to elastic mechanics, the elastic constitutive equations:

(2) $$\begin{array}{c} \varepsilon_{x}=\frac{1}{E}\left[\sigma_{x}-\mu \left(\sigma_{y}+\sigma_{z}\right)\right],\\ \varepsilon_{y}=\frac{1}{E}\left[\sigma_{x}-\mu \left(\sigma_{y}+\sigma_{z}\right)\right],\\ \gamma_{xy}=\frac{2\left(1+\mu \right)}{E}\tau_{xy},\\ \gamma_{yz}=\frac{2\left(1+\mu \right)}{E}\tau_{yz},\\ \gamma_{zx}=\frac{2\left(1+\mu \right)}{E}\tau_{zx}, \end{array}$$

where *E* is elasticity modulus and *μ* is Poisson's ratio.

According to the Mises strength criterion, the equivalent stress on casings under triaxial state of stress is

(3) $$\begin{array}{c} \sigma_{e}=\sqrt{\frac{1}{2}\left[{\left(\sigma_{1}-\sigma_{2}\right)}^{2}+{\left(\sigma_{2}-\sigma_{3}\right)}^{2}+{\left(\sigma_{3}-\sigma_{1}\right)}^{2}\right]}. \end{array}$$

If *σ*_*e*_ is greater than *σ*_*y*_, the casing will yield. The yield strength is 552 MPa [19].

## 3. Basic Assumption and Three Dimensional Finite Difference Models

In order to study the effect of thickness and elasticity modulus of mudstone interbed on the open wellbore shrinkage and equivalent stress on casing after cementing operation, three-dimensional finite difference models of bedded salt rock-cement ring casing were established. The basic assumptions are as follows.

1. The casing and cement ring are ideal cylinders with uniform thickness and same center.
2. The casing and cement ring are elastic cylinders with transverse isotropy.
3. The interfaces between casing and cement ring and bedded salt rock do not slide.
4. The stratum is distributed horizontally.

**Figure 1:**
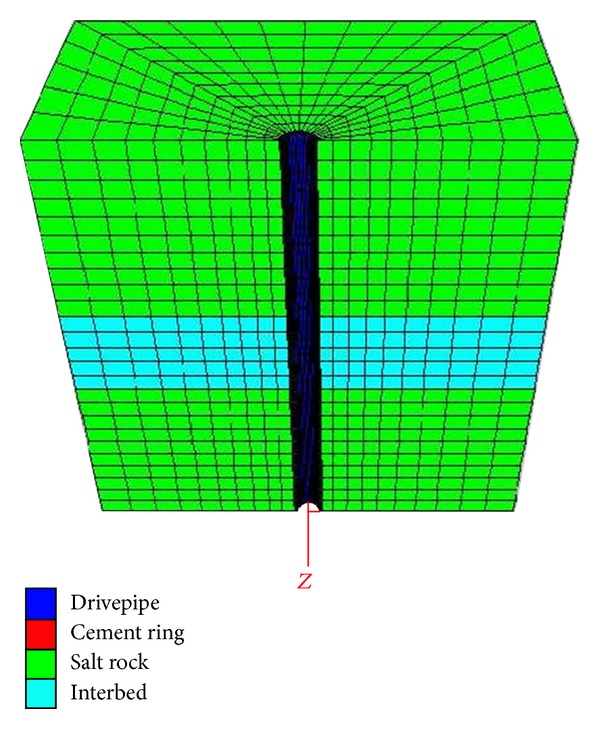
Mesh graph of XOY plane.

**Figure 2:**
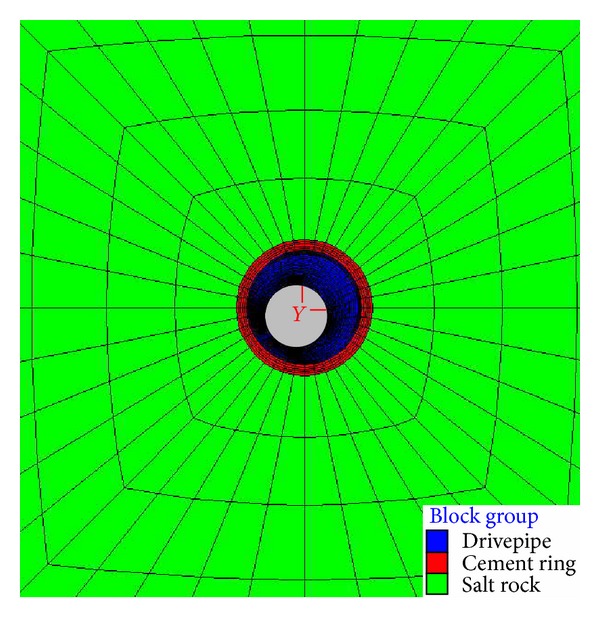
Mesh graph of XOZ plane.

According to the Saint-Venant principle, when the size of model is 6 times larger than the diameter of wellbore, the boundary effect can be ignored. The size of the calculation model is 10 m × 10 m × 20 m. The outside diameter of casing is 273.1 mm, and the thickness is 10.16 mm. The thickness of cement ring is 25 mm. In order to assure the accuracy and efficiency of numerical simulation, the grid near wellbore is intensive. The numerical calculation model is shown in Figures 1 and 2.

The bottom surface and surrounding surfaces have simple supported constraint. The upper surface is covered with crustal stress. The gravity causes the vertical crustal stress. The uniform horizontal crustal stress is *σ*_*H*_ = *σ*_*h*_ = 0.8*σ*_*V*_. Suppose that the stratum gravity is 25 kN/m^3^. The vertical crustal stress is 50 MPa, and horizontal crustal stress is 40 MPa.

The material mechanical parameters of casing, cement ring and salt rock are shown in Table 2. The influence of the thickness and elasticity modulus of the mudstone interbed on open wellbore shrinkage and equivalent stress on casing were analyzed through numerical simulation. In order to control variables, two simulation schemes were designed, which are shown in Tables 3 and 4.

## 4. The Effect of the Thickness and Elasticity Modulus on Open Wellbore Shrinkage

The influence of the thickness of mudstone interbed on open wellbore shrinkage is analyzed in no. 1 simulation scheme. In no. 1 simulation scheme, the mud density is 1.2 g/cm^3^. The radial displacement of shrinkage per unit time calculated through no. 1 simulation scheme is shown in Table 5, from which we find that the radial displacement per unit time decreases along with the increase of mudstone interbed thickness when the elasticity modulus is a constant. When the mudstone interbed thickness increases from 0.2 m to 2.0 m, the radial displacement of shrinkage per unit time in the middle of salt rock decreases from 0.3842 mm to 0.2637 mm, and the radial displacement per unit time in the middle of mudstone interbed decreases from 0.3213 mm to 0.1306 mm. 

The radial displacement of shrinkage per unit time calculated through no. 2 simulation scheme is shown in Table 6. The result shows that when the thickness is unchanging, the radial displacement per unit time decreases along with the increasing elasticity modulus of mudstone interbed. When the elasticity modulus of mudstone interbed increases from 10 GPa to 30 GPa, the radial displacement per unit time in the middle of salt rock decreases from 0.3134 mm to 0.2243 mm, and the radial displacement per unit time in the middle of mudstone interbed decreases from 0.1951 mm to 0.1023 mm.

From Tables 5 and 6, we can see that mudstone interbed whose thickness and elasticity modulus are big can restrain shrinkage effectively and mitigate risk of drill pipe sticking. When the mud density is designed in drilling engineer, thickness and elasticity modulus of mudstone interbed should be comprehensively considered.

## 5. The Effect of the Thickness and Elasticity Modulus on Equivalent Stress on Casing

**Figure 3:**
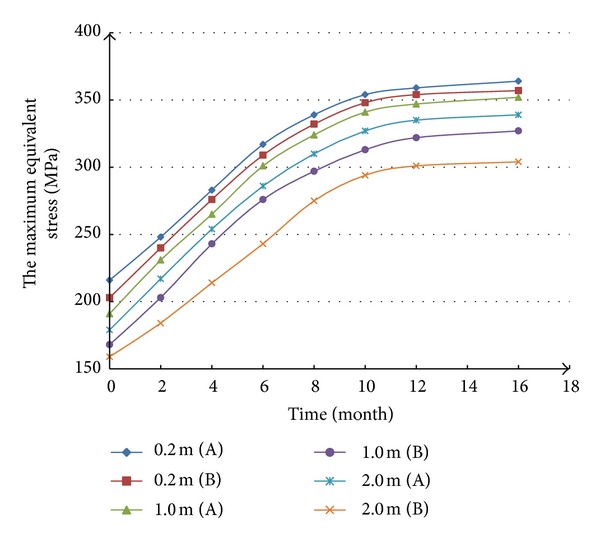
Relationship between the thickness of mudstone interbed and the casing equivalent stress. (A) The casing equivalent stress in the middle of salt rock. (B) The casing equivalent stress in the middle of mudstone interbed.

When the influence of thickness and elasticity modulus of mudstone interbed on casing equivalent stress are analyzed, the internal pressure of casing is ignored. The result of no. 1 simulation scheme is shown in Figure 3, from which we can know that with the increasing of thickness, the equivalent stress of casing in the middle of mudstone interbed decreases, so does the equivalent stress of casing in the middle of salt rock. The equivalent stress in the middle of salt rock is bigger than that of in the middle of the adjacent mudstone interbed. The accident of casing collapse is more likely to happen in the middle of salt rock than in the mudstone interbed. When the thickness of mudstone interbed increases from 0.2 m to 2.0 m, the equivalent stress on casing in the middle of salt rock decreases from 364 MPa to 339 MPa, and the equivalent stress in the middle of mudstone interbed decreases from 357 MPa to 304 MPa. The equivalent stress of casing tends to be stable 12 months later.

**Figure 4:**
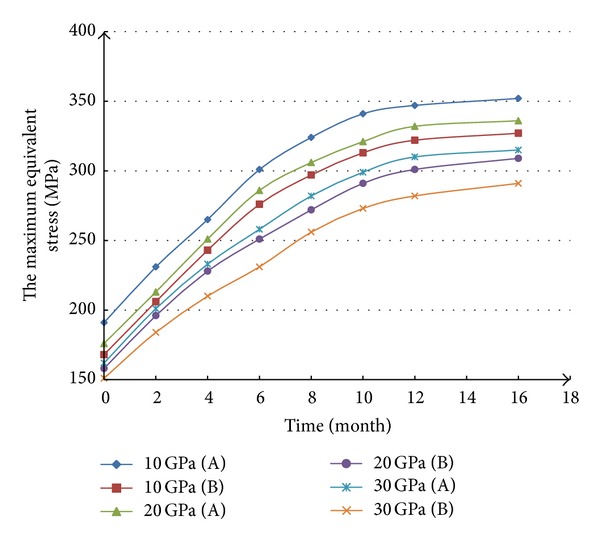
Relationship between the elasticity modulus of mudstone interbed and the equivalent stress on casing. (A) The casing equivalent stress in the middle of salt rock. (B) The casing equivalent stress in the middle of mudstone interbed.

Figure 4 shows the result of No. 2 simulation scheme. With the increasing of elasticity modulus of mudstone interbed, the casing equivalent stress in the middle of mudstone interbed decreases, so does the equivalent stress of casing in the middle of salt rock. When the elasticity modulus of mudstone interbed increases from 10 GPa to 30 GPa, the equivalent stress on casing in the middle of salt rock decreases from 352 MPa to 315 MPa, and the equivalent stress in the middle of mudstone interbed decreases from 327 MPa to 291 MPa. The equivalent stress on casing tends to be basically stable after 12 months.

From Figures 3 and 4, it is clear that the casing in salt rock stratum with mudstone interbed that has small thickness and low elasticity modulus runs a higher risk of casing collapse. When we design the casing strength in drilling engineer, thickness and elasticity modulus of mudstone interbed should be taken into consideration.

## 6. Conclusions

In this paper, the three-dimensional finite difference model of bedded salt rock-cement ring casing was established to analyze the influence of mudstone interbed on open wellbore shrinkage and equivalent stress on casing after cementing operation.

1. With the increase of thickness and elasticity modulus of mudstone interbed, the radial displacement of shrinkage per unit time decreases. Mudstone interbed whose thickness and elasticity modulus are bigger can restrain shrinkage effectively and mitigate risk of drill pipe sticking.
2. With the increase of thickness and elasticity modulus of mudstone interbed, the equivalent stress on casing decreases. The casing in salt rock stratum with mudstone interbed that has small thickness and low elasticity modulus runs a higher risk of casing collapse.
3. The equivalent stress on casing increases with time and becomes stable 12 months later.

## Figures and Tables

**Table 1:** Material creep parameters of salt rock and mudstone interbed.

	Creep parameter *A*	Creep parameter *n*
Salt rock	1.12 × 10^−8^	3.8
Mudstone interbed	5 × 10^−10^	4.35

**Table 2:** Material mechanical parameters.

	Elasticity modulus (GPa)	Poisson's ratio
Salt rock	18	0.3
Cement ring	40	0.24
Casing	210	0.23

**Table 3:** No. 1 simulation scheme.

Name	Thickness (m)	Elasticity modulus (GPa)	Poisson's ratio
Mudstone interbed	0.2	10	0.27
	1.0		
	2.0		

**Table 4:** No. 2 simulation scheme.

Name	Thickness (m)	Elasticity modulus (GPa)	Poisson's ratio
Mudstone interbed	1.0	10	0.27
		20	
		30	

**Table 5:** The radial displacement of shrinkage per unit time under different mudstone interbed thickness (mm).

Shrinkage place	Mudstone interbed thickness (m)		
	0.2	1.0	2.0
Middle of salt rock	0.3842	0.3134	0.2637
Middle of mudstone interbed	0.3213	0.1951	0.1306

**Table 6:** The radial displacement of shrinkage per unit time under different elasticity modulus of mudstone interbed (mm).

Shrinkage place	Elasticity modulus of mudstone interbed (GPa)		
	10	20	30
Middle of salt rock	0.3134	0.2817	0.2243
Middle of mudstone interbed	0.1951	0.1426	0.1023
